# Alcohol-Induced Glycolytic Shift in Alveolar Macrophages Is Mediated by Hypoxia-Inducible Factor-1 Alpha

**DOI:** 10.3389/fimmu.2022.865492

**Published:** 2022-05-11

**Authors:** Niya L. Morris, David N. Michael, Kathryn M. Crotty, Sarah S. Chang, Samantha M. Yeligar

**Affiliations:** ^1^ Department of Medicine, Division of Pulmonary, Allergy, Critical Care and Sleep Medicine, Emory University, Atlanta, GA, United States; ^2^ Atlanta Veterans Affairs Health Care System, Decatur, GA, United States

**Keywords:** ethanol, hypoxia-inducible factor-1 alpha, alveolar macrophage, energy metabolism, glycolysis

## Abstract

Excessive alcohol use increases the risk of developing respiratory infections partially due to impaired alveolar macrophage (AM) phagocytic capacity. Previously, we showed that chronic ethanol (EtOH) exposure led to mitochondrial derangements and diminished oxidative phosphorylation in AM. Since oxidative phosphorylation is needed to meet the energy demands of phagocytosis, EtOH mediated decreases in oxidative phosphorylation likely contribute to impaired AM phagocytosis. Treatment with the peroxisome proliferator-activated receptor gamma (PPARγ) ligand, pioglitazone (PIO), improved EtOH-mediated decreases in oxidative phosphorylation. In other models, hypoxia-inducible factor-1 alpha (HIF-1α) has been shown to mediate the switch from oxidative phosphorylation to glycolysis; however, the role of HIF-1α in chronic EtOH mediated derangements in AM has not been explored. We hypothesize that AM undergo a metabolic shift from oxidative phosphorylation to a glycolytic phenotype in response to chronic EtOH exposure. Further, we speculate that HIF-1α is a critical mediator of this metabolic switch. To test these hypotheses, primary mouse AM (mAM) were isolated from a mouse model of chronic EtOH consumption and a mouse AM cell line (MH-S) were exposed to EtOH *in vitro.* Expression of HIF-1α, glucose transporters (Glut1 and 4), and components of the glycolytic pathway (Pfkfb3 and PKM2), were measured by qRT-PCR and western blot. Lactate levels (lactate assay), cell energy phenotype (extracellular flux analyzer), glycolysis stress tests (extracellular flux analyzer), and phagocytic function (fluorescent microscopy) were conducted. EtOH exposure increased expression of HIF-1α, Glut1, Glut4, Pfkfb3, and PKM2 and shifted AM to a glycolytic phenotype. Pharmacological stabilization of HIF-1α *via* cobalt chloride treatment *in vitro* mimicked EtOH-induced AM derangements (increased glycolysis and diminished phagocytic capacity). Further, PIO treatment diminished HIF-1α levels and reversed glycolytic shift following EtOH exposure. These studies support a critical role for HIF-1α in mediating the glycolytic shift in energy metabolism of AM during excessive alcohol use.

## Introduction

Over 15 million people in the United States have been diagnosed with alcohol use disorders (1). Excessive alcohol use increases morbidity and mortality (2) and increases risk of developing respiratory infections (3), which is largely linked to immune dysfunction in alveolar macrophages (AM) (4–7). AM initiate the immune response to pathogens in the lower airway (8), but excessive alcohol use impairs AM phagocytic capacity and bacterial clearance (5, 9). Phagocytosis requires high energy demands, and mitochondrial-dependent oxidative phosphorylation is the most efficient method of generating cellular ATP. Our laboratory has established that chronic alcohol exposure results in AM mitochondrial dysfunction (e.g., mitochondrial fragmentation, morphological alteration, and derangements in mitochondrial bioenergetics) (10). Further, treatment with the peroxisome proliferator-activated receptor gamma (PPARγ) ligand, pioglitazone (PIO), improved AM phagocytic dysfunction (7, 11) and oxidative phosphorylation (10) during ethanol (EtOH) exposure.

One mechanism employed by cells to meet their energy demands in the absence of oxidative phosphorylation is glycolysis (12). Glycolysis is a metabolic pathway that converts glucose into pyruvate utilizing enzymatic proteins, such as 6-phosphofructo-2-kinase/fructose-2,6-bisphosphatase 3 (Pfkfb3) and pyruvate kinase M2 (PKM2), to generate energy (12, 13). Blocking key glycolytic proteins such as Pfkb3 and PKM2 has been shown to mitigate acute lung injury (14, 15). The effect of EtOH on these glycolytic proteins in AM has not been explored.

Stabilization of hypoxia-inducible factor (HIF)-1α and subsequent formation of HIF-1 (comprised of the inducible HIF-1α and constitutive HIF-1β) increases the transcription of numerous genes including those in the glycolytic pathway, such as glucose transporters (GLUT) 1 and 4 and pyruvate dehydrogenase kinase 1 (PDK-1) (16–19). Mounting evidence suggests that HIF-1α may act as a “metabolic switch”, shifting cells from relying on oxidative phosphorylation towards glycolysis instead (17–19). The availability of glucose needed for glycolysis is in part regulated by glucose transporters which transport glucose into the cell (12). HIF-1α (with GLUT and PDK-1) have been shown in other models to contribute to lung injury (20–22). Further, numerous studies have shown a direct relationship between HIF-1α and EtOH-mediated pathologies in the brain (23), adipose tissue (24), and liver (25). The findings from these studies showed that EtOH-induced HIF-1α can occur during oxidative stress or elevated inflammation.

The relationship between HIF-1α and these metabolic derangements in the context of chronic EtOH-induced AM phagocytic dysfunction, however, have not been examined and are the focus of the current study. Our data demonstrate that HIF-1α is a critical mediator of EtOH-mediated energy derangements in AM, suggesting a key role of HIF-1α in EtOH-mediated lung pathobiology. Further, PIO attenuated EtOH-induced HIF-1α, which could provide a novel therapeutic strategy in the treatment of alcohol use disorders in the lung and decrease susceptibility to respiratory infections.

## Materials and Methods

### Mouse Model of Chronic Ethanol Ingestion

Animal studies were carried out in accordance with the National Institutes of Health guidelines as outlined in the *Guide for the Care and Use of Laboratory Animals*. Additionally, all protocols were reviewed and approved by the Atlanta VA Health Care System Institutional Animal Care and Use Committee. 8- to 10-week-old male C57BL/6J mice purchased from Jackson Laboratory (Bar Harbor, Maine, United States) were fed standard laboratory chow *ad libitum*. Mice were randomly divided into two groups (control and EtOH). EtOH fed mice received increases of EtOH (5% w/v) in their drinking water for 2 weeks until the EtOH concentration reached 20% w/v and this concentration was maintained for 10 weeks, resulting in a 0.12% blood alcohol level (6, 7, 26). During the last week of ethanol ingestion, mice were administered PIO (10 mg/kg/day in 100-µL methylcellulose vehicle) or vehicle alone *via* oral gavage (7). Following euthanasia, tracheas were cannulated, and a tracheotomy was performed to collect bronchoalveolar lavage fluid. Bronchoalveolar lavage fluid was centrifuged at 8000 RPM for 5 minutes to isolate mouse alveolar macrophages (mAM). Isolated mAM were resuspended in RPMI-1640 culture medium (2% fetal bovine serum and 1% penicillin/streptomycin) for 24 hours for further experimentation (6, 7). Lung tissue was harvested and homogenized for RNA isolation.

### *In Vitro* Ethanol Exposure of MH-S Cells

The mouse alveolar macrophage cell line (MH-S) was purchased from American Type Culture Collection (Manassas, VA, United States). MH-S cells were cultured in RPMI-1640 medium (10% fetal bovine serum, 1% penicillin/streptomycin, 11.9 mM sodium bicarbonate, gentamicin (40mg/ml) and 0.05 mM 2- mercaptoethanol) in the presence or absence of 0.08% EtOH for 72 hours (media changed daily) at 37°C in a humidified incubator in 5% CO_2_ (5, 6). In a subset of experiments, MH-S were treated with PIO (10 μM; last 24 hours of EtOH exposure) (Cayman Chemicals, Ann Arbor, Michigan, United States).

### Cell Energy Phenotype Test

Cell energy phenotype tests were performed to evaluate the metabolic phenotypes of mAM and MH-S using either an XFe96 (Catalog number: 103325-100) or an XFp extracellular flux analyzer (Catalog number: 103275-100) (Agilent Seahorse Bioscience Inc.; Billerica, MA, United States). Oxygen consumption rate (OCR) and extracellular acidification rate (ECAR) were measured in mAM and MH-S over time in XF Base Medium supplemented with 1 mM of sodium pyruvate, 10 mM glucose, and 2 mM of L-glutamine followed by a single injection of 2 μM oligomycin (ATP synthase inhibitor) + 0.5 μM carbonilcyanide p-triflouromethoxyphenylhydrazone (FCCP; a mitochondrial uncoupling agent). XFp plates were precoated with collagen (~4 hours) and washed with PBS and media prior to addition of mAM cells to promote mAM adherence to the plates. Raw OCR and ECAR were determined using the XF Wave 2.1 software. OCR and ECAR values were calculated, normalized to cell protein concentration in the same sample, and were expressed as mean of biological replicates ± standard error of the mean (SEM).

### Glycolysis Stress Test

Glycolysis stress tests were performed using either an XFe96 or an XFp extracellular flux analyzer (Agilent Seahorse Bioscience Inc.) to evaluate the parameters of glycolytic flux. ECAR was measured in mAM and MH-S over time in XF Base Medium supplemented with 2 mM L-glutamine followed by sequential injections of 10 mM glucose (saturating concentration of glucose to promote glycolysis), 2 μM oligomycin (ATP synthase inhibitor), and 50 mM 2-deoxy-glucose (2-DG; a glucose analog that inhibits glycolysis). To maximize mAM adherence to XFp microculture plates, wells were precoated with collagen (~4 hours) and were subsequently washed with PBS and media before addition of cells. Glycolysis, glycolytic capacity, glycolytic reserve, and non-glycolytic acidification were determined using the XF Wave 2.1 software. Raw ECAR was determined using the XF Wave 2.1 software. Glycolysis, glycolytic capacity, glycolytic reserve, and non-glycolytic acidification ECAR values were calculated, normalized to cell protein concentration in the same sample, and were expressed as mean of biological replicates ± SEM.

### RNA Isolation and Quantitative RT-PCR (qRT-PCR)

TRIzol reagent (Catalog number:15596026, Invitrogen, Waltham, MA, United States) was used to isolate total RNA. Primer sequences outlined in **Table 1** were used to measure and quantify target mRNA levels by qRT-PCR with iTaq Universal SYBR Green One-Step kit (Catalog number: 1725151, Bio-Rad, Hercules, CA, United States) using the Applied Biosystems ABI Prism 7500 version 2.0.4 sequence detection system (6, 7). Target mRNA values were normalized to 9S or glyceraldehyde 3-phosphate dehydrogenase (GAPDH). mRNA levels were expressed as fold-change of mean ± SEM, relative to control samples.

**Table 1:** Primer sequences to measure mRNA levels using qRT-PCR.

	Gene	Forward Sequence (5’ → 3’)	Reverse Sequence (5’ → 3’)
Mouse	GAPDH	GGATTTGGTCGTATTGGG	GGAAGATGGTGATGGGATT
Mouse	Glut1	CTCCTGCCCTGTTGTGTATAG	AAGGCCACAAAGCCAAAGAT-
Mouse	Glut4	AAAAGTGCCTGAAACCAGAG	TCACCTCCTGCTCTAAAAGG
Mouse	HIF-1α	CTCAAAGTCGGACAG	CCCTGCAGTAGGTTT
Mouse	Pfkfb3	TCTAGAGGAGGTGAGATCAG	CCTGCCACTCTTATCTTCTG
Mouse	Pkm2	GAGGCCTCCTTCAAGTGCT	CCAGACTTGGTGAGGACGAT
Mouse	9S	ATCCGCCAGCGCCATA	TCGATGTGCTTCTGGGAATCC

GAPDH; glyceraldehyde 3-phosphate dehydrogenase, Glut1; glucose transporter 1, Glut4; glucose transporter 4, HIF-1α; hypoxia-inducible factor-1 alpha, Pfkfb3; 6-phosphofructo-2-kinase/fructose-2,6-bisphosphatase 3, PKM2; pyruvate kinase M2.

### Cytoimmunostaining and Phagocytosis by Fluorescent Microscopy

HIF-1α protein was measured in mAM isolated from control- and EtOH-fed mice. mAM were fixed with 4% paraformaldehyde and incubated with a HIF-1α rabbit monoclonal antibody (1:500, Cell Signaling Technology, Danvers, MA, United States) for 1 hour, washed, and incubated with fluorescent-labeled anti-rabbit secondary antibody (1:1000) for 1 hour. Protein values were normalized to DAPI nuclear stain.

*In vitro* phagocytic capacity in MH-S was determined using pHrodo *Staphylococcus aureus* BioParticles conjugate (Catalog number: A10010, Invitrogen). MH-S (1.2 × 10^5^ cells) were incubated with 1 × 10^6^ particles of pH-sensitive fluorescent-labeled *S. aureus* for 2 hours. Following the incubation, cells were fixed with 4% paraformaldehyde. Cells with internalized *S. aureus* were considered positive for phagocytosis. Phagocytic capacity was quantified as phagocytic index: cells positive for internalized bacteria is multiplied by the relative fluorescent units (RFU) of *S. aureus* per cell. Phagocytic index is expressed as fold-change of mean ± SEM, relative to control samples (7, 11).

Fluorescence for HIF-1α cytoimmunostaining and phagocytosis of *S. aureus* was measured using FluoView (Olympus, Melville, New York, United States) and are expressed as fold-change of mean relative fluorescent units RFU per cell ± SEM, relative to control samples. RFU were evaluated in at least 10 cells per field, with 10 fields per experimental condition. Gain and gamma microscope settings were constant for each field and experimental condition. ImageJ was used to deconvolute and analyze images (10, 27).

### Western Blot

Proteins were isolated from MH-S using SESSA lysis buffer and quantified using the Pierce bicinchoninic acid (BCA) Protein Assay Kit (Catalog number for Pierce bicinchoninic acid (BCA) Protein Assay Reagent A: 23228 and Catalog number for Pierce bicinchoninic acid (BCA) Protein Assay Reagent B: 23224, Thermofisher, Waltham, Massachusetts, United States). Equal amounts of protein from cell lysates were loaded on NuPAGE Novex 10% Bis-Tris Protein Gels (Catalog number: NP0301BOX, Fisher Scientific, Hampton, NH, United States) subsequent to being transferred onto nitrocellulose membranes. The membranes were blocked in 5% non-fat milk and TBST for 1 hour and then incubated with primary antibodies for HIF-1α rabbit monoclonal antibody (Catalog number: 14179S, 1:500, Cell Signaling Technology) or glyceraldehyde 3-phosphate dehydrogenase rabbit polyclonal antibody (Catalog number: G9545-100UL, 1:20,000, GAPDH, Sigma-Aldrich, St. Louis, MO, United States) overnight at 4°C. Following this incubation, the membranes were washed and incubated with 1:10,000 anti-rabbit IRDye800CW Secondary Antibodies (Catalog number: 926-32211, Li-COR Biosciences, Lincoln, NE, United States) for 1 hour at room temperature. Odyssey Infrared Imaging System (LI-COR Biosciences) was used to image the membranes. Image J software (NIH, Bethesda, MD, United States) was used to measure densitometry. HIF-1α protein values were normalized to GAPDH and expressed as fold-change of mean ± SEM, relative to control samples.

### Lactate Assay

Lactate levels in MH-S were determined using a lactate assay kit (Catalog number: MAK064, Sigma Aldrich) according to the manufacturer’s instructions. Lactate values were normalized to protein concentration in the same sample and were expressed as fold-change of mean ± SEM, relative to control samples.

### Cobalt Chloride Treatment of MH-S

MH-S were treated with the HIF-1α stabilizer cobalt (II) chloride hexahydrate (Catalog number: C8661-25g, 25 μM, CoCl_2_, Sigma-Aldrich) in PBS vehicle or PBS alone for 4 hours. CoCl_2_ increases HIF-1α expression (28) and stabilizes HIF-1α by inhibiting the binding of von Hippel Lindau E3 ubiquitin ligase, preventing HIF-1α ubiquitination and subsequent degradation (29).

### Transient Transfection of MH-S

HIF-1α was silenced in MH-S using transient transfection of a HIF-1α siRNA (Catalog number: sc-35562, Santa Cruz, Dallas, TX, United States), and HIF-1α was induced in MH-S using transient transfection of HIF-1α lysate (Catalog number: sc-120778, Santa Cruz). MH-S were resuspended in 100 μL of Amaxa Mouse Macrophage Nucleofector Kit solution (Catalog number: VPA-1009, Lonza, Alpharetta, GA, United States) containing 100 nM of control scrambled (Catalog number: sc-37007, control-scr, Santa Cruz), siRNA for HIF-1α (siHIF-1α), or HIF-1α lysate (HIF-1α) followed by nucleofection according to the manufacturer’s protocol using program Y-001. Following transfection, MH-S were washed with media and cultured with or without 0.08% EtOH for 3 days (media changed daily).

### Statistical Analysis

Data are presented as mean ± SEM. A Student’s t-test was used in studies with two groups. In studies, with more than two groups, statistical significance was calculated using one-way analysis of variance (ANOVA) followed by Tukey-Kramer *post hoc* (GraphPad Prism version 9, San Diego, CA). In the event that the data was not normally distributed, a non-parametric statistical analysis using Kruskal-Wallis test was used. p<0.05 was considered significant.

## Results

### Ethanol Shifted AM to a Glycolytic Metabolic Phenotype

**Figure 1:**
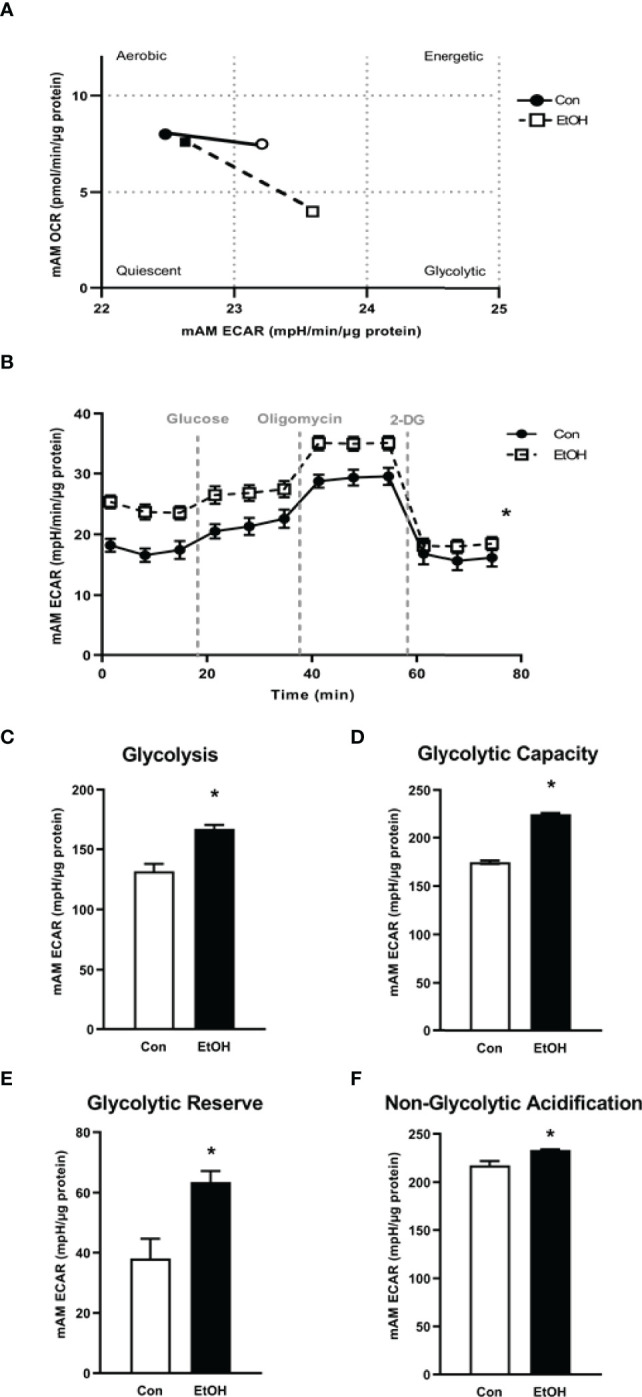
EtOH induces glycolysis in mAM. Mouse alveolar macrophages (mAM) were isolated from mice fed either control (Con) or ethanol (EtOH; 20% v/w in drinking water, 12 weeks). **(A)** Oxygen consumption rates (OCR) and extracellular acidification rates (ECAR) were measured in response to an injection mixture of oligomycin (oligo; mitochondrial complex V inhibitor) and carbonilcyanide p-triflouromethoxyphenylhydrazone (FCCP; ATP synthase inhibitor and proton uncoupler) using an extracellular flux analyzer. Cell energy phenotype was measured, normalized to protein levels, and are expressed as mean ± SEM (n = 4-5). ECAR were measured in response to sequential injections of glucose (saturating concentration of glucose to promote glycolysis), oligomycin (ATP synthase inhibitor), and 2-deoxy-glucose (2-DG; a glucose analog that inhibits glycolysis) using an extracellular flux analyzer. OCR measures are pmol over time and ECAR measures are mpH over time, normalized to total protein in the same sample well, and are expressed as mean ± SEM. Parameters of glycolytic function **(B)**, glycolysis **(C)**, glycolytic capacity **(D)**, glycolytic reserve **(E)**, and non-glycolytic acidification **(F)** are expressed as mean ± SEM, relative to control (n = 12-14). *p < 0.05 verses control.

**Figure 2:**
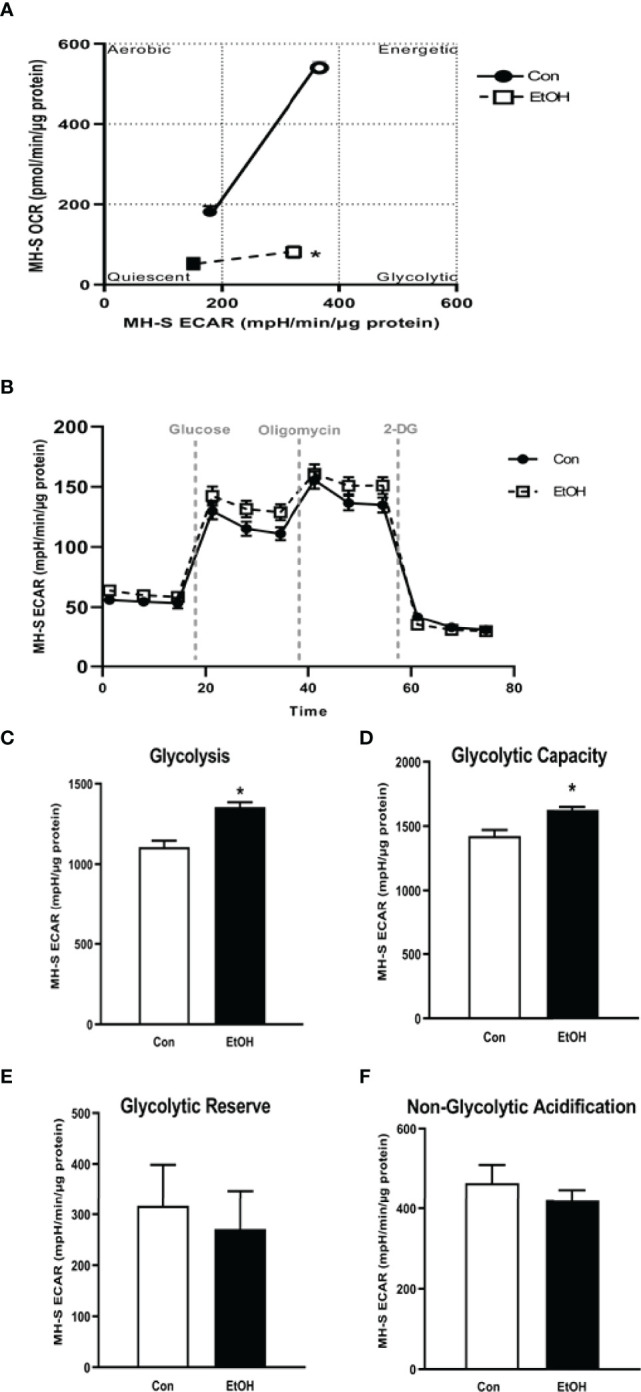
EtOH induces glycolysis in MH-S cells. MH-S were exposed to either control (Con) or ethanol (EtOH; 0.08%) for 72 hours. **(A)** Oxygen consumption rates (OCR) and extracellular acidification rates (ECAR) were measured in response to an injection mixture of oligomycin (oligo; mitochondrial complex V inhibitor), carbonilcyanide p-triflouromethoxyphenylhydrazone (FCCP; ATP synthase inhibitor and proton uncoupler) using an extracellular flux analyzer. Cell energy phenotype was measured, normalized to protein levels, and are expressed as mean ± SEM (n = 3). ECAR were measured in response to sequential injections of glucose (saturating concentration of glucose to promote glycolysis), oligomycin (ATP synthase inhibitor), and 2-deoxy-glucose (2-DG; a glucose analog that inhibits glycolysis) using an extracellular flux analyzer. OCR measures are pmol over time and ECAR measures are mpH over time, normalized to total protein in the same sample well, and are expressed as mean ± SEM. Parameters of glycolytic function **(B)**, glycolysis **(C)**, glycolytic capacity **(D)**, glycolytic reserve **(E)**, and non-glycolytic acidification **(F)** are expressed as mean ± SEM, relative to control (n = 6). *p < 0.05 verses control.

Previously, we have shown that EtOH exposure altered mitochondrial morphology and negatively impacted mitochondrial bioenergetics (10). To assess whether EtOH exposure increased glycolysis, we evaluated the cell energy phenotype of mAM isolated from control and EtOH-fed mice. mAM from EtOH-fed mice shifted to a glycolytic phenotype in response to the oligomycin +FCCP stressors (**Figure 1A**). To provide further evidence that EtOH resulted in glycolytic shift, we performed a glycolysis stress test on mAM from control and EtOH fed mice. Compared with mAM from control mice, mAM from EtOH fed mice exhibited increased glycolytic profiling (**Figure 1B**), glycolysis (**Figure 1C**), glycolytic capacity (**Figure 1D**), glycolytic reserve (**Figure 1E**), and non-glycolytic acidification (**Figure 1F**). Similar to our *in vivo* studies, glycolytic bioenergetics were elevated in EtOH-treated MH-S (**Figure 2**) compared to control. Assessment of the cell energy phenotype of EtOH treated MH-S exhibited a glycolytic shift compared to control (**Figure 2A**). Additionally, EtOH treated MH-S displayed increased glycolytic profiling compared to control (**Figure 2B**). Finally, glycolysis (**Figure 2C**), and glycolytic capacity (**Figure 2D**) were also elevated in EtOH-treated MH-S compared to controls. We did not observe any differences in glycolytic reserve (**Figure 2E**) or non-glycolytic acidification (**Figure 2F**) between the groups. Collectively, these data illustrate that AM exhibit a glycolytic energy phenotype in response to EtOH.

### Ethanol Increased Glycolytic Proteins in Mouse Lungs and MH-S

**Figure 3:**
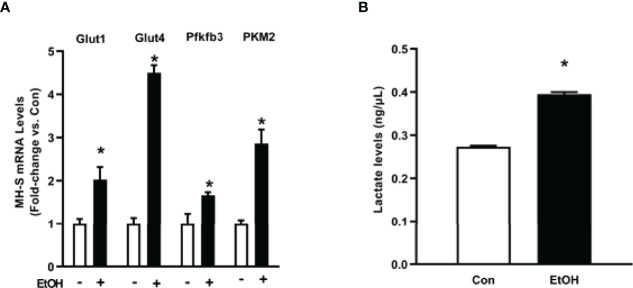
Ethanol increases expression of glycolytic proteins and lactate levels in MH-S. MH-S were exposed to either control (Con) or ethanol (EtOH; 0.08%) for 72 hours. **(A)** mRNA levels of glucose transporter (Glut)1, Glut4, 6-phosphofructo-2-kinase/fructose-2,6-bisphosphatase 3 (Pfkfb3), and pyruvate kinase 2 (PKM2) were measured by qRT-PCR, in duplicate, normalized to GAPDH, and are expressed as mean ± SEM, relative to control. **(B)** Protein isolated from MH-S cells was used to evaluate lactate levels *via* lactate assay kit and are expressed as mean ± SEM, relative to control (n = 4-6). *p < 0.05 versus control.

As we observed increases in glycolytic flux following EtOH exposure in AM, we assessed expression of the glucose transporters, Glut1 and Glut4, and key enzymes of the glycolytic pathway, Pfkfb3 and PKM2. mRNA levels of Glut1, Glut4, Pfkfb3, and PKM2 were increased in response to EtOH (**Figure 3A**). Additionally, EtOH induced mRNA expression of Glut1 in mouse lung homogenates (**Supplementary Figure 1**). Since lactate levels correlate with generation of ECAR during glycolysis (30), we investigated the effect of EtOH on AM lactate levels. Lactate was elevated in response to EtOH in MH-S (**Figure 3B**). These results further suggest that EtOH induces glycolysis in mouse lungs and AM.

### Ethanol-Induced HIF-1α in mAM and MH-S

**Figure 4:**
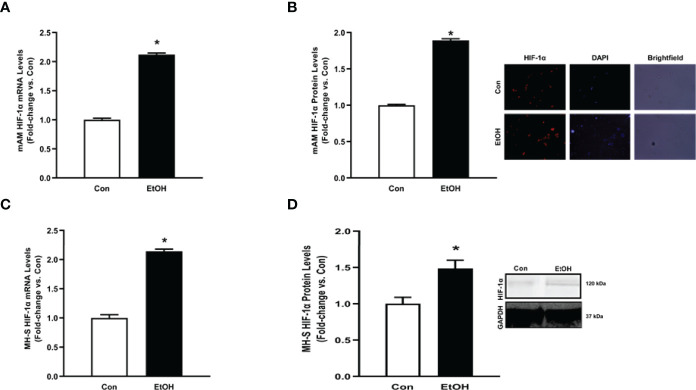
Ethanol induces HIF-1α in mAM and MH-S. **(A, B)** Mouse alveolar macrophages (mAM) were isolated from mice fed either control (Con) or ethanol (EtOH; 20% v/w in drinking water, 12 weeks). **(A)** HIF-1α mRNA levels were measured by qRT-PCR, in duplicate, normalized to 9S, and expressed as mean ± SEM, relative to control. **(B)** HIF-1α protein levels were measured by fluorescence microscopy (10 fields/condition), normalized to DAPI, and are expressed as mean RFU ± SEM, relative to control. Representative microscopy images have been provided. **(C, D)** MH-S were exposed to either control (Con) or ethanol (EtOH; 0.08%) for 72 hours. **(C)** HIF-1α and were measured by qRT-PCR, in duplicate, normalized to GAPDH, and expressed as mean ± SEM, relative to control (n = 6). **(D)** HIF-1α protein levels were evaluated *via* western blot, normalized to GAPDH protein, and densitometry is expressed as mean ± SEM, relative to control (n = 4). Representative western blot images have been provided. *p < 0.05 versus control.

We sought to investigate the mechanism by which EtOH increased parameters of glycolytic flux in AM. HIF-1α, a component of the transcription factor HIF-1, can act as a “metabolic switch”. HIF-1 increases the transcription of some genes in the glycolytic pathway and has been shown in other models to be increased by EtOH exposure (23–25, 31). Here, we examined how EtOH affected hypoxia-inducible factor (HIF)-1α in AM. mRNA and protein levels of HIF-1α were measured in control and EtOH mAM. EtOH feeding elevated mAM HIF-1α mRNA (**Figure 4A**) and protein (**Figure 4B**) expression. Similarly, we observed increases in HIF-1α mRNA (**Figure 4C**) and protein (**Figure 4D**) in MH-S exposed to EtOH compared to control. Collectively, these data show that EtOH induces HIF-1α in AM.

### Ethanol-Induced Derangements in AM Glycolytic Shift Is Mediated by HIF-1α in MH-S

**Figure 5:**
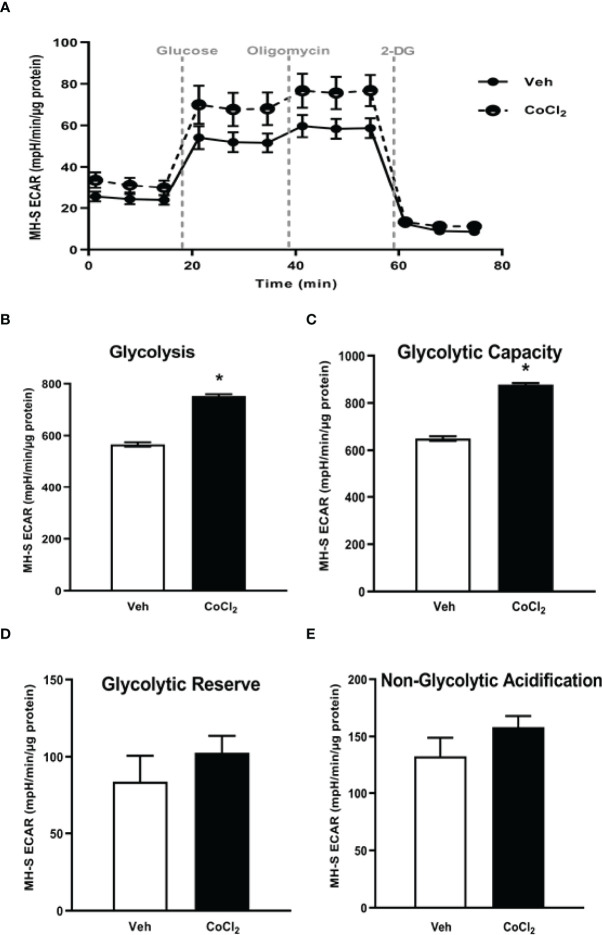
Stabilization of HIF-1α *in vitro via* cobalt chloride mimics EtOH-mediated derangements in MH-S. MH-S were exposed to either vehicle (Veh) or cobalt chloride (CoCl_2_, 25 μM) for 4 hours. Extracellular acidification rates (ECAR) were measured in response to sequential injections of glucose (saturating concentration of glucose to promote glycolysis), oligomycin (ATP synthase inhibitor), and 2-deoxy-glucose (2-DG; a glucose analog that inhibits glycolysis) using an extracellular flux analyzer. ECAR from glycolytic profiling, normalized to protein levels, and are expressed as mean ± SEM **(A)**, glycolysis **(B)**, glycolytic capacity **(C)**, glycolytic reserve **(D)**, and non-glycolytic acidification **(E)** are expressed as mean ± SEM, relative to vehicle (n = 6). *p < 0.05 versus vehicle.

**Figure 6:**
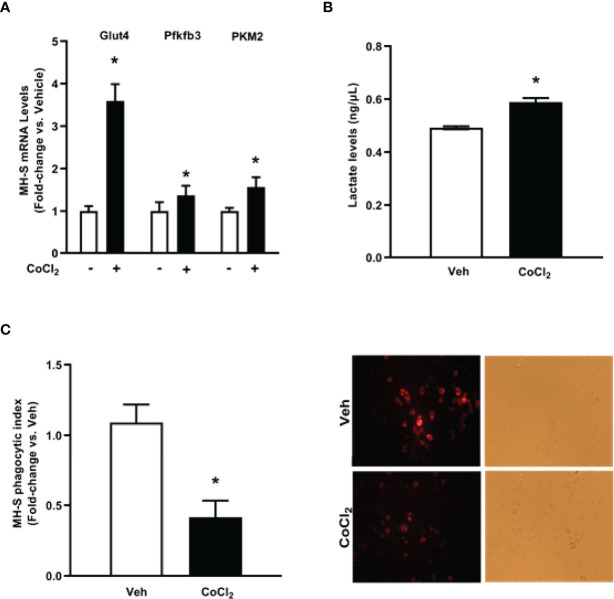
Cobalt chloride induces expression of glycolytic proteins and lactate levels and causes phagocytic dysfunction in MH-S. MH-S were exposed to either vehicle (Veh) or cobalt chloride (CoCl_2_, 25 μM) for 4 hours. **(A)** mRNA levels of glucose transporter (Glut)4, 6-phosphofructo-2-kinase/fructose-2,6-bisphosphatase 3 (Pfkfb3), and pyruvate kinase 2 (PKM2) were measured by qRT-PCR, in duplicate, normalized to GAPDH, and are expressed as mean ± SEM, relative to control (n = 4-6). **(B)** Protein isolated from MH-S was used to evaluate lactate levels *via* lactate assay kit and are expressed as mean ± SEM, relative to vehicle (n = 6). *p < 0.05 versus vehicle. **(C)** Phagocytic index was calculated from the percentage of cells positive for bacterial uptake multiplied by the RFU of *S. aureus* per cell. Values are expressed as mean ± SEM relative to vehicle (n = 5). Representative fluorescent and brightfield images have been provided. *p < 0.05 versus vehicle.

To establish whether HIF-1α is implicated in EtOH-mediated glycolytic shift in AM, control MH-S were treated with cobalt chloride, a HIF-1α stabilizer. Treatment of MH-S with cobalt chloride mimicked the increase in HIF-1α mRNA (**Supplementary Figure 2A**) and protein (**Supplementary Figure 2B**) seen in AM exposed to EtOH (**Figure 4**). Cobalt chloride exposed MH-S exhibited increases in components of glycolytic profiling (**Figure 5A**), glycolysis (**Figure 5B**), and glycolytic capacity (**Figure 5C**) similar to our EtOH studies of AM (**Figures 1**, **2**). Similar to our *in vitro* studies (**Figure 2**), we did not observe changes in glycolytic reserve (**Figure 5D**) and non-glycolytic capacity (**Figure 5E**) with cobalt chloride treatment. Concomitantly, treatment of MH-S with HIF-1α lysate increased glycolytic profiling (**Supplementary Figure 3A**), glycolysis (**Supplementary Figure 3B**), glycolytic capacity (**Supplementary Figure 3C**), and glycolytic reserve (**Supplementary Figure 3D**). Glut4, Pfkfb3, and PKM2 (**Figure 6A**) mRNA levels and lactate levels (**Figure 6B**) were increased in response to cobalt chloride, similar EtOH-treated MH-S (**Figure 3**). As cobalt chloride is a mimetic for HIF-1α, these data suggest that EtOH-induced HIF-1α mediates the glycolytic shift observed in AM. Further, similar to our EtOH studies (7, 11), treatment with cobalt chloride led to AM phagocytic dysfunction (**Figure 6C**).

### HIF-1α Modulates EtOH-Induced Glycolysis and Phagocytic Function in MH-S

**Figure 7:**
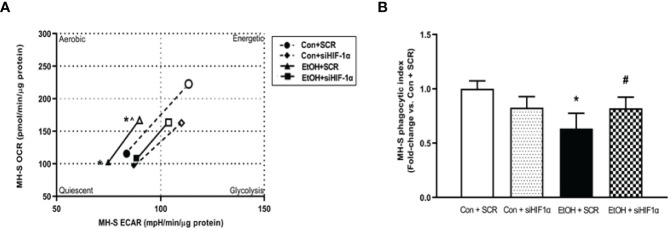
HIF-1α modulates EtOH-induced glycolysis and phagocytic function in MH-S. MH-S transiently transfected with control scramble (SCR) or siRNA against HIF-1α (siHIF1α) were exposed to either Con or EtOH (0.08%) for 72 hours. **(A)** Oxygen consumption rates (OCR) and extracellular acidification rates (ECAR) were measured in response to an injection mixture of oligomycin (oligo; mitochondrial complex V inhibitor) and carbonilcyanide p-triflouromethoxyphenylhydrazone (FCCP; ATP synthase inhibitor and proton uncoupler) using an extracellular flux analyzer. Cell energy phenotype was measured, normalized to protein levels, and are expressed as mean ± SEM (n = 5). ECAR were measured in response to sequential injections of glucose (saturating concentration of glucose to promote glycolysis), oligomycin (ATP synthase inhibitor), and 2-deoxy-glucose (2-DG; a glucose analog that inhibits glycolysis) using an extracellular flux analyzer. OCR measures are pmol over time and ECAR measures are mpH over time, normalized to total protein in the same sample well, and are expressed as mean ± SEM. **(B)** Phagocytic index was calculated from the percentage of cells positive for bacterial uptake multiplied by the RFU of *S. aureus* per cell. Values are expressed as mean ± SEM relative to vehicle (n = 6). *p < 0.05 versus control; ^#^ *p* < 0.05 versus ethanol.

To further implicate HIF-1α in modulating EtOH-induced glycolysis, we knocked down HIF-1α in the presence and absence of EtOH. We determined that knockdown of HIF-1α prevented EtOH-mediated glycolytic shift (**Figure 7A**). Further, these improvements coincided with improved phagocytic index in MH-S lacking HIF-1α in the presence of EtOH (**Figure 7B**). Collectively, these data show that HIF-1α plays a key role in EtOH-mediated increases in AM glycolysis and impaired phagocytic capacity.

### Pioglitazone Treatment Reverses Ethanol-Induced HIF-1α

**Figure 8:**
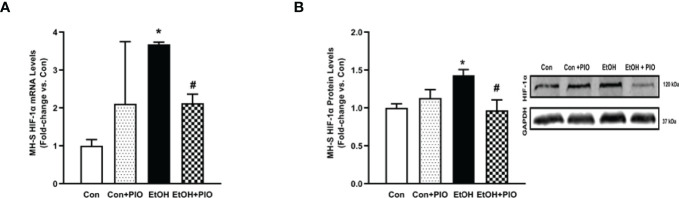
Pioglitazone treatment reverses EtOH-induced HIF-1α levels. MH-S exposed to either control (Con) or ethanol (EtOH; 0.08%) for 72 hours ± pioglitazone (PIO; 10 μM, last 24 hours of EtOH exposure). **(A)** HIF-1α mRNA levels were measured by qRT-PCR, in triplicate, normalized to GAPDH, and expressed as mean ± SEM, relative to control (n = 3). *p < 0.05 versus control; ^#^ *p* < 0.05 versus EtOH. **(B)** HIF-1α protein levels were evaluated *via* western blot, normalized to GAPDH protein, and densitometry is expressed as mean ± SEM, relative to control (n  = 3). Representative western blot images have been provided. *p < 0.05 versus control; ^#^ *p* < 0.05 versus ethanol.

The PPARγ ligand, PIO, has been previously reported to improve EtOH-mediated mitochondrial derangements (10), and phagocytic dysfunction (7, 11). As such, we sought to delineate whether PIO may affect EtOH-induced AM HIF-1α. PIO treatment diminished HIF-1α mRNA (**Figure 8A**) and protein (**Figure 8B**) levels. Collectively, these data identify PIO as a therapeutic strategy to mitigate EtOH-induced HIF-1α in AM.

### Pioglitazone Treatment Reverses EtOH-Induced Glycolysis

**Figure 9:**
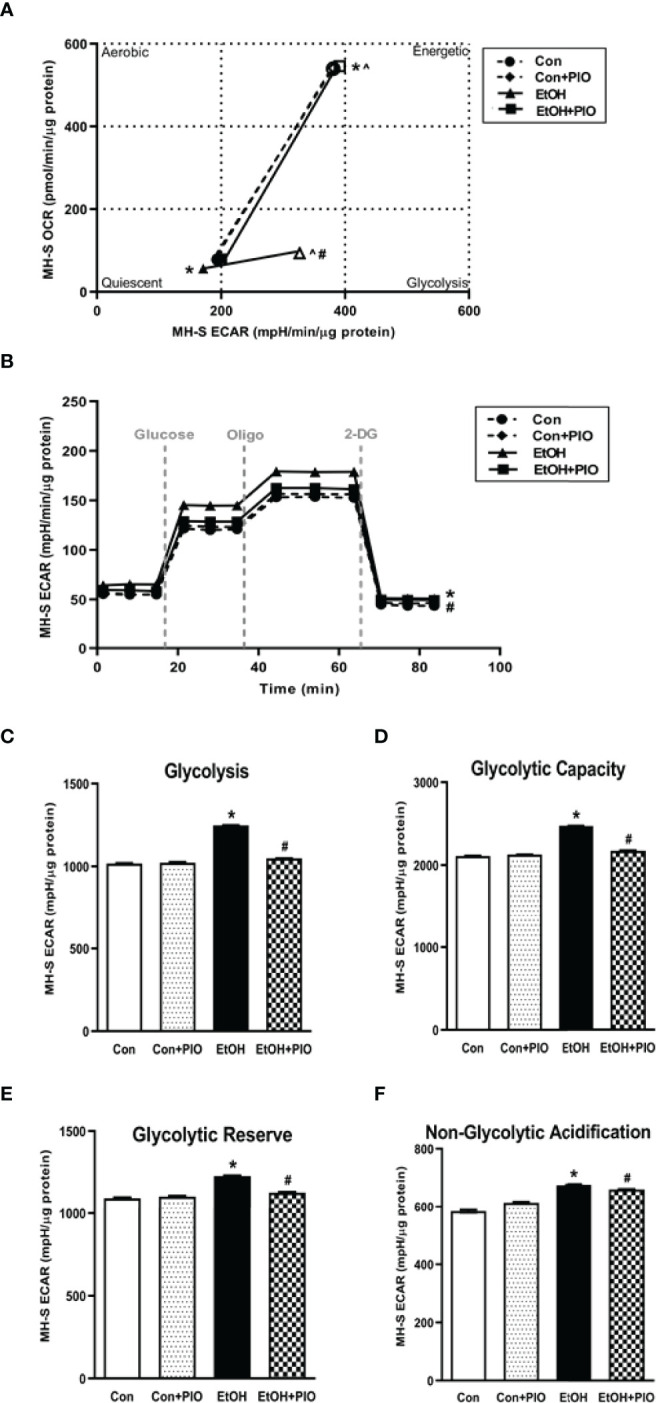
Pioglitazone treatment reverses EtOH-induced glycolysis in MH-S. MH-S were exposed to either control (Con) or ethanol (EtOH; 0.08%; 72 hours) ± pioglitazone (PIO, last day of ethanol). **(A)** Oxygen consumption rates (OCR) and extracellular acidification rates (ECAR) were measured in response to an injection mixture of oligomycin (oligo; mitochondrial complex V inhibitor), carbonilcyanide p-triflouromethoxyphenylhydrazone (FCCP; ATP synthase inhibitor and proton uncoupler) using an extracellular flux analyzer. Cell energy phenotype was measured, normalized to protein levels, and are expressed as mean ± SEM (n = 15). ECAR were measured in response to sequential injections of glucose (saturating concentration of glucose to promote glycolysis), oligomycin (ATP synthase inhibitor), and 2-deoxy-glucose (2-DG; a glucose analog that inhibits glycolysis) using an extracellular flux analyzer. OCR measures are pmol over time and ECAR measures are mpH over time, normalized to total protein in the same sample well, and are expressed as mean ± SEM. Parameters of glycolytic function **(B)**, glycolysis **(C)**, glycolytic capacity **(D)**, glycolytic reserve **(E)**, and non-glycolytic acidification **(F)** are expressed as mean ± SEM, relative to control (n = 15). *p < 0.05 verses control; ^#^ *p* < 0.05 versus ethanol; ^*p* < 0.05 versus control stressed.

**Figure 10:**
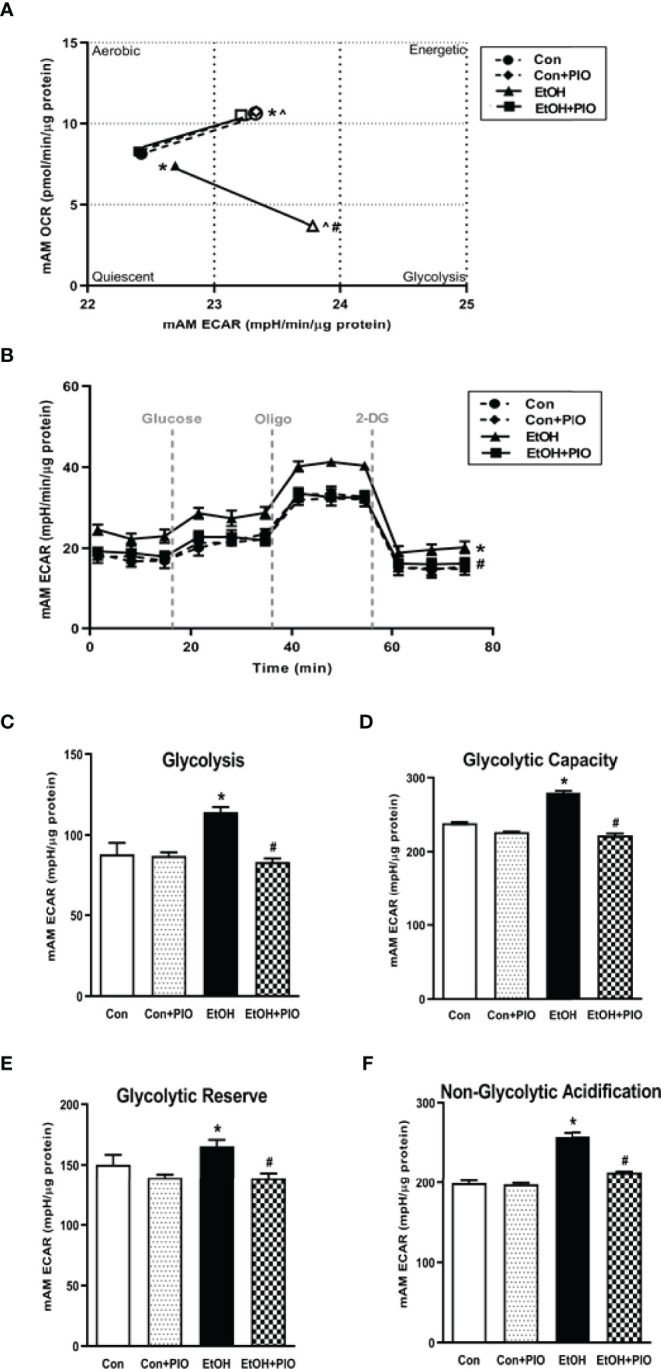
Pioglitazone treatment reverses EtOH-induced glycolysis in mAM. Mouse alveolar macrophages (mAM) were isolated from mice fed either control (Con) or ethanol (EtOH; 20% v/w in drinking water) ± oral pioglitazone (PIO, last 7 days of ethanol). **(A)** Oxygen consumption rates (OCR) and extracellular acidification rates (ECAR) were measured in response to an injection mixture of oligomycin (oligo; mitochondrial complex V inhibitor) and carbonilcyanide p-triflouromethoxyphenylhydrazone (FCCP; ATP synthase inhibitor and proton uncoupler) using an extracellular flux analyzer. Cell energy phenotype was measured, normalized to protein levels, and are expressed as mean ± SEM (n = 10-12). ECAR were measured in response to sequential injections of glucose (saturating concentration of glucose to promote glycolysis), oligomycin (ATP synthase inhibitor), and 2-deoxy-glucose (2-DG; a glucose analog that inhibits glycolysis) using an extracellular flux analyzer. OCR measures are pmol over time and ECAR measures are mpH over time, normalized to total protein in the same sample well, and are expressed as mean ± SEM. Parameters of glycolytic function **(B)**, glycolysis **(C)**, glycolytic capacity **(D)**, glycolytic reserve **(E)**, and non-glycolytic acidification **(F)** are expressed as mean ± SEM, relative to control (n = 11-14). *p < 0.05 versus control; ^#^ *p* < 0.05 versus ethanol; ^*p* < 0.05 versus control stressed.

As treatment with PIO improved mitochondrial derangements due to EtOH exposure (10), here we sought to determine if PIO affected glycolysis in MH-S in the presence of EtOH. As demonstrated previously, EtOH induced a glycolytic shift in response to oligomycin+FCCP stressors however, PIO treatment prevented the EtOH-induced glycolytic shift in MH-S (**Figure 9A**). Treatment with PIO also reversed EtOH-induced increases in the MH-S glycolytic bioenergetics parameters, glycolytic profiling (**Figure 9B**), glycolysis (**Figure 9C**), glycolytic capacity (**Figure 9D**), glycolytic reserve (**Figure 9E**), and non-glycolytic acidification (**Figure 9F**). Similarly, PIO treatment prevented the glycolytic shift in mAM isolated from EtOH-fed mice (**Figure 10A**). Treatment with PIO also reversed EtOH-induced increases in the mAM glycolytic bioenergetics parameters, glycolytic profiling (**Figure 10B**), glycolysis (**Figure 10C**), glycolytic capacity (**Figure 10D**), glycolytic reserve (**Figure 10E**), and non-glycolytic acidification (**Figure 10F**). Collectively, these data show that AM glycolytic energy phenotype in response to EtOH is reversed with PIO treatment.

## Discussion

One of the hallmark immune functions of AM is to phagocytose invading pathogens in the lower respiratory tract (8). In order to meet the high energy demands of phagocytosis, oxidative phosphorylation is the most efficient process utilized for cellular ATP generation. Previously, we have demonstrated that EtOH exposure severely diminishes the ability of AM to phagocytose and clear pathogens (4–7). Further, we have shown that EtOH altered mitochondria morphology and diminished oxidative phosphorylation in MH-S. Additionally, we demonstrated that the PPARγ ligand, PIO, partially reversed EtOH-induced AM mitochondrial derangements (10) and improved EtOH-induced AM phagocytic dysfunction (11). However, the mechanisms by which EtOH alters AM metabolism have not been fully elucidated. This study aimed to evaluate whether HIF-1α has a role in EtOH-mediated energy derangements in AM. Our findings provide evidence that EtOH shifts AM to a glycolytic metabolic phenotype, which is mediated by EtOH-induced HIF-1α. Also, PIO treatment diminishes EtOH-induced HIF-1α, providing HIF-1α as a molecular mechanism by which PIO improves AM phagocytic function. This study establishes HIF-1α as a critical modulator of chronic EtOH-mediated metabolic derangements in AM.

This study provides a mechanistic understanding of our previous study (10) by showing that EtOH-mediated decreases in oxidative phosphorylation is due to a glycolytic shift. One method of meeting the metabolic requirements of the cell in the absence of oxidative phosphorylation is glycolysis. Glucose transporters transport glucose into the cell, providing some of the glucose needed for glycolysis (12). Glycolysis is a multistep process which utilizes proteins such as Pfkfb3 and PKM2 (12, 13). Our findings herein show that EtOH increases glycolysis (**Figures 1**, **2**). The variance in EtOH-induced alterations in ECAR in mAM (**Figure 1B**) versus MH-S (**Figure 2B**) may be due to the difference in duration of EtOH exposure (mAM isolated from mice fed EtOH for 12 weeks versus MH-S exposed to 0.08% EtOH *in vitro* for 72 hours) and systemic, physiological effects of EtOH. However, the glycolysis bioenergetics profiles for glycolysis and glycolytic capacity were comparable between these mAM *in vivo* (**Figures 1C, D**) and MH-S *in vitro* (**Figures 2C, D**) models. Further, we observed mRNA levels of glucose transporters (GLUT1 and GLUT 4) were elevated following EtOH exposure (**Figure 3**). Further, EtOH induced Pfkfb3, PKM2, and lactate in AM (**Figure 3**). Together, these data demonstrate that EtOH shifts AM to a glycolytic phenotype.

Other studies have described a direct relationship between HIF-1α and EtOH-mediated pathologies (23–25, 31). These studies have demonstrated that EtOH-induced HIF-1α occurs under conditions of elevated inflammation or oxidative stress. Other models have investigated the role of HIF-1α in chronic lung injury (20, 21). HIF-1α was activated *in vitro* in human pulmonary artery smooth muscle cells, demonstrating a role of HIF-1α in pulmonary hypertension pathogenesis (20). HIF-1α has been branded a “metabolic switch”, shifting cells from utilizing oxidative phosphorylation to glycolysis (17–19). However, the relationship between HIF-1α and metabolic derangements in the context of chronic EtOH-induced AM phagocytic dysfunction have not been established until now and are supported by the data presented herein. This study illustrates that chronic EtOH exposure increases HIF-1α expression (**Figure 4**). Further, as shown in **Figures 5**, **6**, treatment with the HIF-1α mimetic, cobalt chloride, causes AM derangements similar to EtOH. Knockdown of HIF-1α in the presence of EtOH prevented EtOH induced glycolytic shift and glycolytic profiling (**Figures 7A, B**). Taken together, these data suggest that HIF-1α is a critical modulator of EtOH-induced glycolytic phenotype in AM. Interestingly, Kang et al. showed that EtOH did not alter glycolysis in bone marrow derived macrophages. The group did, however, conclude that EtOH increased glycolytic capacity, glycolytic reserve, and non-glycolytic acidification. HIF-1α expression and activity was also increased due to EtOH exposure (32). The slight variance in results between our studies could be due to the differences in experimental models using bone marrow-derived macrophages to model the AM phenotype. AM may be tissue-resident or recruited cells with key differential functions in host defense (33). However, the current study provides evidence of the critical role for HIF-1α in mediating the glycolytic shift in AM due to EtOH exposure using an AM cell line and AM isolated from *in vivo* EtOH-fed mice. As HIF-1α is a component of the transcription factor HIF-1; elevated levels could have effects not related to glycolysis. One limitation of the current study is that it does not explore non glycolytic effects of HIF-1α. As described above, previous reports have shown that HIF-1α is elevated as a response to inflammation or oxidative stress (23–25, 31), and our lab has shown that oxidative stress contributes to AM phagocytic impairments (7, 10, 11). Modulation of HIF-1α could be alleviating EtOH-mediated oxidative stress, thus improving phagocytic dysfunction.

Since HIF-1 is a transcription factor with numerous targets, other targets may be of future interest. For example, the HIF-1 target PDK-1 can repress mitochondrial function and oxygen consumption. PDK-1-mediated phosphorylation inhibits pyruvate dehydrogenase, preventing the use of pyruvate in oxidative phosphorylation and resulting in decreased mitochondrial oxygen consumption (34). Additionally, other mechanisms, such as fatty acid oxidation, may be involved in meeting the energy demands of the cell due to EtOH exposure. However, studies in the liver suggest that chronic alcohol exposure promotes hepatic injury but does not increase the rate of fatty acid β-oxidation through inhibition of mitochondrial β-oxidation (35–37).

Previously, our lab has shown that alcohol-mediated decreases in peroxisome proliferator-activated receptor gamma (PPARγ) cause AM dysfunction (7). PPARγ is activated by synthetic ligands, such as PIO. This results in heterodimerization of PPARγ with a retinoid receptor and subsequent binding to the PPAR response element in the promoter region of its target genes. The response to this binding is dependent on whether the heterodimerization results in recruitment of coactivators (increases gene expression) or corepressors (decreases gene expression) (38). Our lab has shown that treatment with PPARγ ligands diminished oxidative stress following chronic EtOH exposure (7, 10, 11). Interestingly, decreased expression of PPARγ impaired AM phagocytic capacity following chronic EtOH exposure (7). However, the mechanism by which PPARγ mediates these effects is not known. Other models which generate reactive oxygen species (ROS) have determined that there is an inverse relationship between PPARγ and HIF-1α and that PPARγ ligand treatment decreased hypoxia-induced HIF-1α expression (20, 39). Here, we show that treatment PIO attenuated EtOH-induced HIF-1α (**Figure 8**). It is unclear however, if PPARγ mediates its action on HIF-1α in a direct (binding to HIF-1α promoter) or indirect (reduction of ROS) manner. As shown in **Figures 5**, **6**, the HIF-1α mimetic, cobalt chloride produced results similar to EtOH-induced metabolic derangements. Collectively, these data demonstrated that EtOH-mediated phagocytic dysfunction is in part linked to increased HIF-1α levels, which is mitigated with PIO treatment. Further, PIO treatment reversed EtOH-induced glycolytic bioenergetics (**Figures 9**, **10**).

The current study fills a gap in knowledge by providing a mechanistic understanding to earlier studies which demonstrate that chronic EtOH exposure results in phagocytic dysfunction (4–7, 10) and decreases oxidative phosphorylation (10) in AM. Together, our previous studies suggest that AM has diminished phagocytic capacity due to an inability to meet the energy requirements for phagocytosis. Using both *in vitro* and *in vivo* approaches, we identified HIF-1α as a critical mediator of EtOH-mediated metabolic derangements in AM. These studies establish HIF-1α as a potential therapeutic target for PIO (approved for clinical use in the treatment of type 2 diabetes), which could mitigate the risk of developing respiratory infections in people with a history of alcohol use disorders.

## Data Availability Statement

The original contributions presented in the study are included in the article/**Supplementary Material**. Further inquiries can be directed to the corresponding author.

## Ethics Statement

The animal study was reviewed and approved by Atlanta Veterans Affairs Health Care System Institutional Animal Care and Use Committee.

## Author Contributions

NLM designed experiments, obtained samples from animal experiments, analyzed experiments, and prepared the manuscript; DNM, KMC, and SSC obtained samples from animal experiments and analyzed experiments; SMY designed and analyzed experiments and prepared the manuscript. All authors contributed to the article and approved the submitted version.

## Funding

This work was supported in part by grants from the National Institute on Alcohol Abuse and Alcoholism (R01AA026086) to SMY (ORCID ID: 0000-0001-9309-0233) and the National Heart, Lung, and Blood Institute (T32HL116271) to David M. Guidot, Lou Ann S. Brown, and C. Michael Hart. The contents of this report do not represent the views of the Department of Veterans Affairs or the US Government.

## Conflict of Interest

The authors declare that the research was conducted in the absence of any commercial or financial relationships that could be construed as a potential conflict of interest.

## Publisher’s Note

All claims expressed in this article are solely those of the authors and do not necessarily represent those of their affiliated organizations, or those of the publisher, the editors and the reviewers. Any product that may be evaluated in this article, or claim that may be made by its manufacturer, is not guaranteed or endorsed by the publisher.
