# Supplementary figures and images for: Alcohol-Induced Glycolytic Shift in Alveolar Macrophages Is Mediated by Hypoxia-Inducible Factor-1 Alpha
Source: Front Immunol. 2022 May 11;13:865492. doi: 10.3389/fimmu.2022.865492 (PMC9130492; doi:10.3389/fimmu.2022.865492)

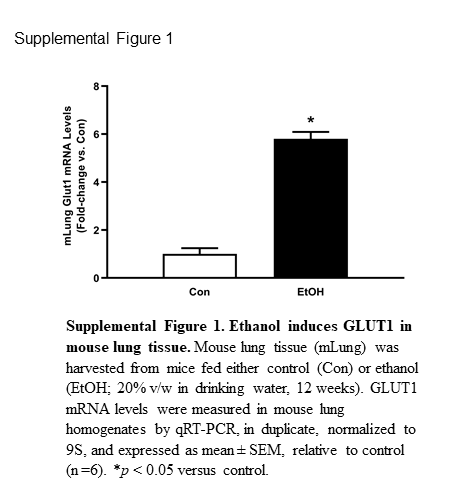

Supplement: Supplementary file 1 [file Image_1.tif]

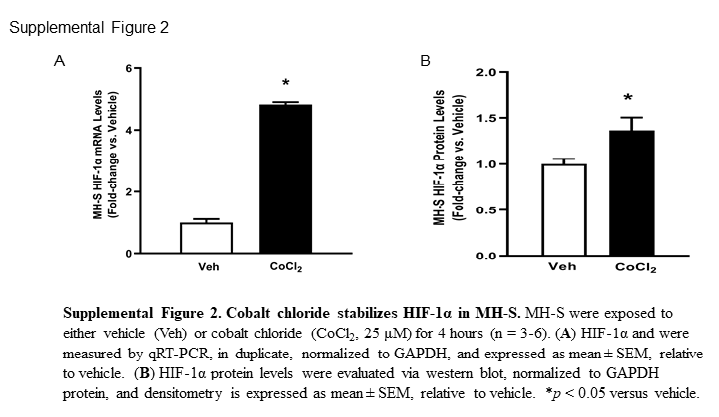

Supplement: Supplementary file 2 [file Image_2.tif]

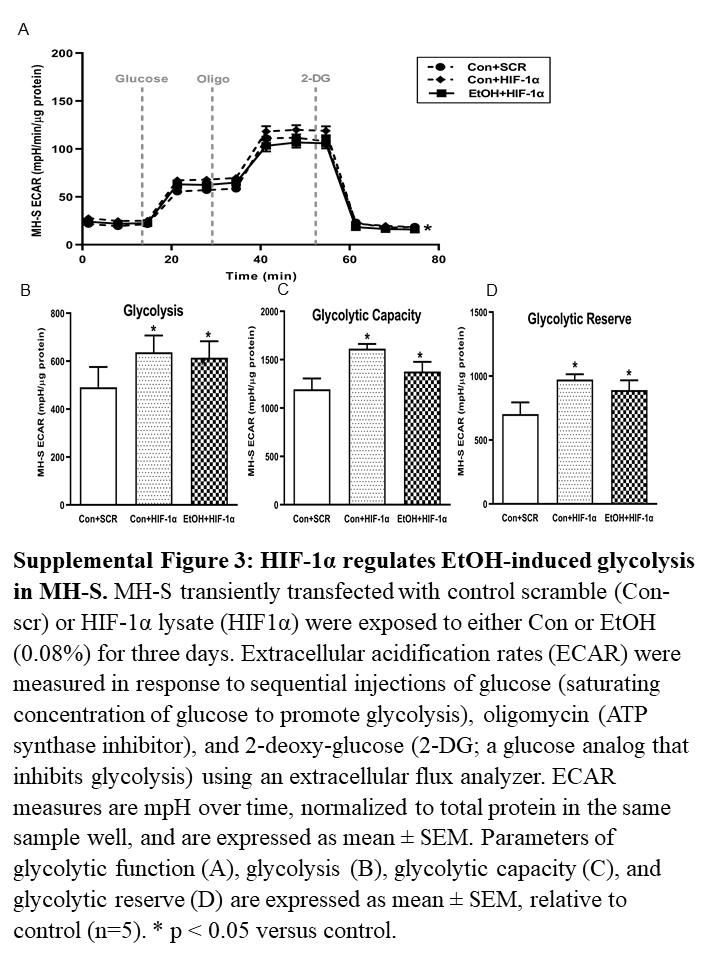

Supplement: Supplementary file 3 [file Image_3.tif]
